# Venereal Diseases, Their Complications and Sequelæ

**Published:** 1900-09

**Authors:** 


VENEREAL DISEASES, THEIR COMPLICATIONS AND SEQUELÆ. By EDWARD L. KEYES, A. M., M. D., Late Professor of Dermatology and Genito-urinary Surgery in the Bellevue Hospital Medical College; and CHARLES H. CHETWOOD, M. D., Professor of Genito-urinary Surgery in the New York Polyclinic College and Hospital. One volume 8vo. New York: William Wood & Co. 1900. [Price, extra muslin, $2.75 net.]

The field of genito-urinary literature appears to be fairly well supplied now, with this volume following so closely on the appearance of Lydston's work, on the same general subject.

The original of the volume under consideration was written nearly twenty years ago by Dr. Keyes, and it was then accepted as the authority. The present issue is a revision. That portion of Dr. Keyes's work relating to syphilis has been changed little if any. In fact, his teachings in the early days regarding the management of syphilis have been adhered to and there have been few additions made to his systematic treatment.

One needs little more than Dr. Keyes's teachings on syphilis to get pretty thoroughly over the ground and obtain a fairly good working idea of that phase of the trouble. Much of his writings on other branches of the general subject have of necessity, by reason of the advancement in treatment and diagnosis, been gone over, and to that extent improved.

On opening the book one senses a feeling of satisfaction for it starts off with the bacteriology of acute specific urethritis. Here, the text describes most minutely and most graphically the appearance and growth of the gonococcus of Neisser, and there is laid down in the minutest manner the process of staining for microscopical determination of the presence of the organism. The illustrating here is particularly good, and admirably true. Colored plates show the various steps taken with the Gram stain, and everything is made plain, very plain, and instructive. The one criticism, which can honestly be made of the whole work, crops out in the following section, that devoted to the general subject of urethritis. Here, the construction is somewhat hazy at times, and in spots the meaning is not quite clear; yet, while this is unfortunate, it cannot be called a fault for it in no wise mars the consideration of the whole subject and its very comprehensive summing up. In those chapters devoted to circumcision one finds some very excellent instruction. The whole subject is considered in detail and the various steps of the operation and the after-treatment are made remarkably clear. Should a person who had never done a circumcision carefully study the text and illustrations, it is safe to say that were he possessed of ordinary intelligence and receptive faculties, he would be able to perform a very creditable bit of work according to the Keyes-Chetwood standard, which is a good one. Throughout instruments mentioned in the text are illustrated clearly; that is, there are no stock wood cuts, which make so many medical volumes look like cheap catalogues. As a whole, the book is a very good, every-day aid to genito-urinary work, as it should be done these modern days. Were I a captious critic, I might take exception to the lack of vehemence regarding urethral sepsis, but that, while good as a general proposition, is largely a matter of opinion. Only works should be criticised. Opinions should be subject for argument—friendy argument.

The best of Dr. Keyes's work has been retained in its original form. These portions in which the progress of medicine has made changes necessary have been brought up to date by Dr. Chetwood with wisdom, care and common sense. N. W. W.

